# Heart Failure Remote Monitoring: A Review and Implementation How-To

**DOI:** 10.3390/jcm12196200

**Published:** 2023-09-26

**Authors:** Elizabeth A. Kobe, Todd McVeigh, Ishaque Hameed, Marat Fudim

**Affiliations:** 1 Department of Medicine, Division of General Internal Medicine, Duke University Medical Center, Durham, NC 27710, USA; 2 Department of Medicine, Division of Cardiology, Duke University Medical Center, Durham, NC 27710, USA; 3 Department of Medicine, DOW University of Health Sciences, Karachi 74200, Pakistan; 4 Duke Clinical Research Institute, Durham, NC 27710, USA

**Keywords:** heart failure, remote monitoring, implementation

## Abstract

Heart failure (HF) is a significant clinical and financial burden worldwide. Remote monitoring (RM) devices capable of identifying early physiologic changes in decompensation have the potential to reduce the HF burden. However, few trials have discussed at length the practical aspects of implementing RM in real-world clinical practice. The present paper reviews current RM devices and clinical trials, focusing on patient populations, outcomes, data collection, storage, and management, and describes the implementation of an RM device in clinical practice, providing a pragmatic and adaptable framework.

## 1. Introduction

Heart failure (HF) represents an increasing clinical, social, and financial burden worldwide. The global prevalence of heart failure is estimated to be 64 million, including 6.7 million in the United States (U.S.) [1,2,3]. In 2012, the economic cost of HF within the U.S. was estimated to be USD 30.7 billion, with projections to increase to USD 69.8 billion by 2030 [4,5]. Similarly, the rates of HF hospitalizations (HFH) and HF readmissions per 1000 U.S. adults in 2017 was 4.9 and 1.1, respectively, a trend that overall seemed to be increasing since 2013 [6].

Avoiding decompensation leading to HFH, however, is challenging. Traditional approaches to monitoring HF patients include frequent weight monitoring, blood pressure measurements, Holter monitoring, or symptom assessments using structured telephone encounters or telemonitoring-based platforms [7,8]. However, these methods are time- and resource-intensive for both patients and providers. Additionally, it has long been known that these parameters often represent later changes in the path of decompensation. A decrease in cardiac output first causes activation of neurohumoral reflexes, leading to the retention of salt and water through the renin–angiotensin–aldosterone system and nonosmotic vasopressin release [9]. The retention of salt and water then leads to increased pulmonary artery pressure, increased interstitial pulmonary fluid manifesting as reduced thoracic impedance, and eventually weight gain and symptoms. Remote monitoring (RM) systems capable of identifying early physiological changes in decompensation have the potential to reduce HFH.

RM devices can collect physiologic data through a variety of mechanisms, including implantable pulmonary artery, left atrial, and inferior vena cava pressure monitoring devices; implantable subcutaneous cardiac monitors; cardiovascular implantable electronic device (CIED)-based technologies; wearable devices; and even stethoscope-based technologies and speech-recognition mobile applications. This manuscript provides an overview of such devices and key clinical studies, with a particular focus on patient populations, outcomes, and, when available, how studies handled RM data from a data management and patient care perspective. We further the current literature surrounding RM devices by additionally describing the real-world implementation of one such device in clinical practice, providing a practical and adaptable framework.

## 2. Remote Monitoring Devices

### 2.1. Implantable Pulmonary Artery Pressure (PAP) Monitoring Devices

**Figure 1:**
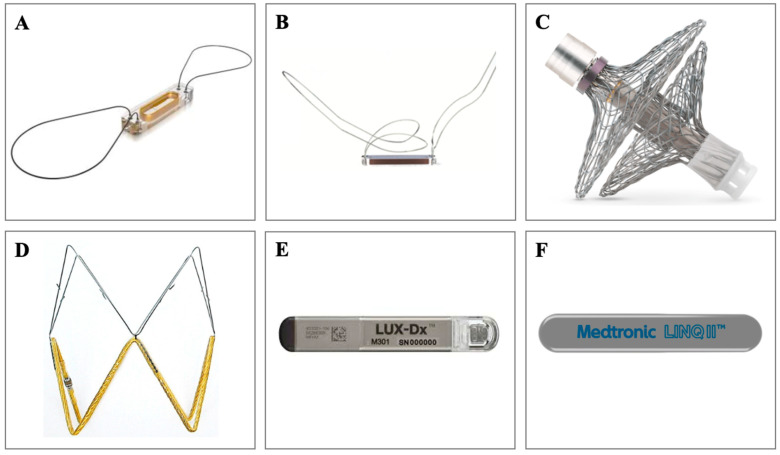
Implantable Devices. (**A**) CardioMEMS (Abbott, Atlanta, GA, USA), (**B**) Cordella (Endotronix, Lisle, IL, USA), (**C**) V-LAP (Vectorious Medical Technologies, Tel Aviv, Israel), (**D**) FIRE1 (FIRE1 Foundry, Dublin, Ireland), (**E**) LUX-Dx (Boston Scientific, Marlborough, MA, USA), (**F**) Reveal LINQ ICM (Medtronic, Minneapolis, MN, USA).

Table 1 summarizes key RM trials for implantable devices and Figure 1 depicts the devices. The CardioMEMS Heart Sensor Allows Monitoring of Pressures to Improve Outcomes in NYHA Functional Class III Heart Failure Patients (CHAMPION) was a prospective, multicenter, randomized, single-blind clinical trial of 550 patients with NYHA class III HF that examined the effectiveness of frequent pulmonary artery pressure (PAP) monitoring on reducing HF-related hospitalizations [10]. CardioMEMS (Abbot) is a coil and pressure-sensitive capacitor that sits in the pulmonary artery. In CHAMPION, patients were assigned to CardioMEMS implantation (treatment group) or standard, consensus-recommended HF management (control group). Patients in the treatment group were instructed to take pulmonary artery pressure measurements daily with the CardioMEMS pillow, which were then automatically transmitted via telephone (landline or cellular) to a secure Internet-based database. Pulmonary artery systolic, diastolic, and mean pressures were used to classify patients as optivolemic (euvolemic), hypervolemic, or hypovolemic. Investigators used daily trends to form these designations and used corresponding guidelines for management. CardioMEMS reduced HF hospitalizations (hazard ratio (HR) 0.72; 95% CI, 0.59–0.88; *p* = 0.0013) but did not reduce mortality (HR 0.68; 95% CI, 0.45–1.02; *p* = 0.06) [11].

In a postapproval study, CardioMEMS reduced HF hospitalizations at 12 months compared to the year before implantation (HR 0.43; 95% CI, 0.39–0.47; *p* < 0.0001) [12]. Like the initial CHAMPION trial, data were transmitted daily, and standardized guidelines were used for management based on daily trends.

GUIDE-HF extended investigation to those with NYHA Class II–IV HF, which confirmed the previous findings of reduced HF hospitalizations with PAP monitoring (HR 0.72; 95% CI, 0.57–0.92; *p* = 0.0072) and also reduced the primary endpoint of HF hospitalizations/urgent visits and all-cause mortality at 12 months (HR 0.81; 95% CI, 0.66–1.00; *p* = 0.049) [13,14].

MEMS-HF extended the investigation into Europe, which again confirmed a reduction in HF hospitalizations (HR 0.38; 95% CI, 0.31–0.48; *p* < 0.0001) and met coprimary outcomes of freedom from device- or system-related complications and from sensor failure [15]. In MEMS-HF, patients recorded daily PAP measurements, which were uploaded to a secure website and reviewed at least weekly, or more frequently if a predefined threshold was automatically triggered by the system, by study personnel and then acted upon by predefined algorithms.

Similarly, MONITOR-HF continued the investigation outside the U.S. in the Netherlands, which demonstrated a reduction in HFH (HR 0.56; 95% CI, 0.38–0.84; *p* = 0.0053), an improvement in quality of life as measured by the Kansas City Cardiomyopathy Questionnaire (KCCQ) (7.13 points; 95% CI, 1.51–12.75; *p* = 0.013), and freedom from device- or system-related complications and from sensor failure [16].

Cordella (Endotronix) is also a direct PAP-monitoring device that is combined with a patient management platform (PMP), a secure cloud-based platform. Initial studies have demonstrated both patient safety and accuracy in mean PAP monitoring with the Cordella Endotronix device (SIRONA and SIRONA II, Endotronix, Inc., Lisle, IL, USA) [17,18].

A prospective, open-label, single-arm, multicenter clinical trial is underway to establish the effectiveness of the Cordella PA Sensor System in NYHA Class III HF (PROACTIVE-HF, NCT04089059). In the trial, daily PAP measurements in addition to weight, blood pressure, heart rate, oxygenation saturation, and symptoms are recorded on a patient management platform for health care providers to review and manage. Clinicians are expected to review data at least once every 4 days, and 7-day PAP averages are used to make guideline-directed management decisions based on concurrent vital sign information, unlike in prior CardioMEMS studies [19].

### 2.2. Implantable Left Atrial Pressure (LAP) Monitoring Devices

Additional implantable remote monitoring devices that directly measure LAP are also underway. The ongoing VECTOR-HF trial is examining an implantable interatrial septum sensor (V-LAP (Vectorious)) capable of transmitting left atrial pressure data via a gateway unit to a secured Cloud Storage database to be reviewed by a medical team. Current data for V-LAP suggest safety, agreeability with wedge pressure measurements, and effectiveness in improving NYHA functional class status [20,21].

### 2.3. Implantable Inferior Vena Cava (IVC) Monitoring Device

Other implantable RM devices are currently being developed for IVC monitoring. The FIRE1 system (FIRE1) is one such device that is implanted between the renal and hepatic veins and transmits IVC respiratory variation in cross-sectional dimensions wirelessly via a wearable belt. Evaluation of the device among 20 sheep demonstrated no device-related complications, no significant differences in IVC area at stable volume status, and significantly increased IVC area with volume infusion [22]. To further expand, experiments conducted compared the sensitivity of IVC area change with changes in cardiac filling pressures and found IVC area changes to be more sensitive during colloid infusion (*p* < 0.001), vasodilation (*p* < 0.001), and cardiac dysfunction induced by rapid pacing (*p* = 0.02) [23]. These studies overall demonstrated the safety and sensitivity of the FIRE1 system, which lays the groundwork for the FUTURE-HF trial (NCT04203576), an ongoing trial in humans that will evaluate the feasibility and safety of implanting the FIRE1 system in stable HF patients.

### 2.4. Insertable Cardiac Monitors (ICMs)

ICMs are leadless subcutaneous devices that continuously monitor heart rhythm data. The LUX-Dx TRENDS Study is an ongoing, multicenter study designed to collect diagnostic sensor data from the LUX-Dx ICM (Boston Scientific) and compare them to reference clinical testing data and heart failure decompensation events among patients with NYHA class II and III HF (NCT04790344). ALLEVIATE-HF is an ongoing prospective, randomized, controlled trial investigating the safety and efficacy of the Reveal LINQ ICM (Medtronic) with a RAMware download and diagnostic-based risk stratification algorithm in guiding care of patients with NYHA class II and III HF (NCT04452149).

### 2.5. Cardiovascular Implantable Electronic Device (CIED) Monitoring

Table 2 summarizes key RM trials for CIEDs. CIEDs’ capabilities have expanded beyond defibrillation and cardiac resynchronization therapy to the collection of detailed data about cardiac function, including surrogates for volume status, such as heart sound intensity, heart rate variability, intrathoracic impedance, and thoracic dielectric sensing, or markers of HF prodromal symptoms, such as increased ectopic activity, low percentage of biventricular pacing, or change in patient activity [24,25,26]. These data can be used remotely in real-time monitoring of acute heart failure decompensation. FAST was a prospective study of 156 HF patients examining the relative sensitivity and unexplained detection rate of changes in intrathoracic impedance using OptiFlow (Medtronic) compared to monitoring changes in weight [27]. Sensitivity was greater (76% versus 23%; *p* < 0.0001) and unexplained detection rate was lower (1.9 versus 4.3 patients/year; *p* < 0.0001) for intrathoracic impedance monitoring compared to changes in daily weight at 60 days. The OptiLink HF trial was a prospective, randomized trial that compared early detection of fluid overload by OptiVol (Medtronic) monitoring with standard clinical care in 1002 patients with NYHA class II and III HF [28]. In the treatment arm, fluid status alerts were automatically linked to a wireless CareAlert text notification to the clinician, which were managed based on a detailed interventional algorithm, including triggering a patient call within 2 business days to discuss symptoms. Fluid status alerts did not significantly improve the composite outcome of all-cause death and cardiovascular hospitalization [29].

Several studies have examined CIED-based diagnostic algorithms, utilizing multiple CIED monitoring parameters. PARTNERS-HF was a prospective, nonrandomized, multicenter observational study that evaluated the utility of a CIED-based diagnostic algorithm in its ability to predict HF hospitalizations among 694 patients [30]. Patients with a positive diagnostic algorithm (based on monthly reviews of device diagnostics) had a 5.5-fold increased risk of HF hospitalization within the next month (HR 5.5; 95% CI, 3.4–8.8, *p* < 0.0001). This study suggested that increased frequency of device data reviews results in increased predictive ability to identify patients at risk for HF hospitalization (HR 3.1 when reviewed quarterly, HR 5.5 when reviewed monthly, and HR 6.9 when reviewed semimonthly), though confidence intervals were not included for all time periods. In REM-HF, 1650 patients were randomized to either CIED weekly RM or usual care [31]. In the treatment group, RM clinical management procedures were formalized in a procedural handbook based on RM trends. There was no significant difference between CIED RM and usual care in the primary outcome of death from any cause or unplanned hospitalization for cardiovascular reasons (RM group 42.4% versus control group 40.8%; *p* = 0.87).

MULTISENSE was a cohort study of 900 patients aimed at developing and validating an alert algorithm (HeartLogic from Boston Scientific) using heart sounds, respiration, thoracic impedance, heart rate, and activity from CIEDs [32]. The HeartLogic algorithm detected HF exacerbations with 70% sensitivity (95% CI, 55.4–82.1), and median time from alert onset to HF exacerbation was 34.0 days (IQR: 19.0 to 66.3 days).

Similarly, SELENE HF was a cohort study of 918 patients aimed at developing and validating a predictive algorithm (Seattle HF Model from Biotronik, Berlin, Germany) for HFH using temporal trends and variability of daily heart rates, arrhythmia burden, physical activity, and thoracic impedance from CIEDs [33]. The Seattle HF Model detected HF hospitalizations with 65.5% sensitivity (95% CI, 45.7–82.1), and median time from alert to HFH was 42 days (IQR: 21–89 days).

A patient-level pooled analysis of TRUST, ECOST, and IN-TIME compared Biotronik CIED-based RM with daily transmissions in usual care [34]. Biotronik RM reduced absolute risk of death at 1 year by 1.9% (95% CI, 0.1–3.8; *p* = 0.037) and composite outcome of all-cause mortality or hospitalization for HF exacerbation by 5.6% (95% CI, 1.5–9.7; *p* = 0.007).

### 2.6. Wearable Devices

**Figure 2:**
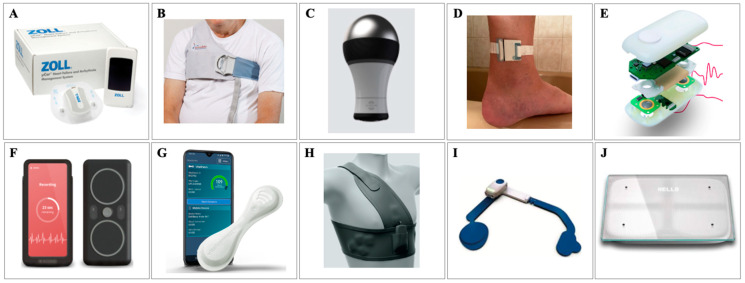
Wearable/noninvasive devices. (**A**) Zoll HFMS (Zoll Medical, Chelmsford, MA, USA), (**B**) ReDS (Sensible Medical, Netanya, Israel), (**C**) Audicor (Inovise Medical, Beaverton, OR, USA), (**D**) BodiGuide Edema Monitor (BodiGuide Inc., Bellevue, DC, USA), (**E**) CardioTag (Cardiosense Inc., Chicago, IL, USA), (**F**) Acorai Heart Monitor (Acorai, Skane, Sweden), (**G**) VitalPatch (Vital Connect, San Jose, CA, USA), (**H**) SimpleSense (Nanowear, Brooklyn, NY, USA), (**I**) Sensinel System (Analog Devices, Wilmington, MA, USA), (**J**) Bodyport Cardiac Scale (Bodyport, San Francisco, CA, USA).

Less invasive, nonimplantable devices are available for the monitoring of both cardiac and extracardiac parameters to detect impending signs of HF exacerbations. Figure 2 depicts these wearable, noninvasive devices. One new, ground-breaking technology is the Zoll HFMS (Zoll Medical), an FDA-approved, wearable, patch-based sensor that uses novel radiofrequency technology to measure thoracic fluid for early detection of changes in pulmonary fluid levels [35]. The Zoll HFMS is capable of recording, storing, and transmitting thoracic fluid data as well as heart rate, respiratory rate, activity, posture, and heart rhythm. Benefits of µCor in Ambulatory Decompensated Heart Failure, or BMADHF, is an international, multicenter, prospective clinical control trial investigating the ability of the Zoll HFMS to reduce recurrent HFH [35]. In BMADHF, patient data reports were automatically sent when thoracic fluid index remained above a set threshold for three consecutive days, prompting clinical teams to act. The study found that the Zoll HFMS reduced recurrent HFH at 90 days compared to control (HR 0.62; *p* = 0.03), which corresponded to an absolute risk reduction of 7% and number needed to treat (NNT) of 14.3 [35]. Looking at a 90-day composite outcome of recurrent HFH, ER visits, or death, the Zoll HFMS resulted in an overall reduction of 38% (HR 0.62; *p* = 0.02), which corresponded to an absolute risk reduction of 9%. Additionally, the Zoll HFMS improved quality of life as measured by KCCQ-12 by an average of 12-points compared to control (*p* = 0.004).

Remote dielectric sensing (ReDS, Sensible Medical) is a noninvasive vest that measures the dielectric properties of tissues, which are mainly determined by lung fluid volume, by using a low-power electromagnetic radar beam. ReDS was designed to aid in volume assessment after discharge to guide adjustments to diuretic therapy [36]. SMILE-HF was a prospective, randomized trial examining the effectiveness of postdischarge HF management guided by remote ReDS assessment in preventing recurrent HF rehospitalization among 268 patients [37]. Frequent home ReDS assessment reduced HF readmissions by 48% compared to usual care (HR 0.52; 95% CI, 0.31–0.87; *p* = 0.01).

Audicor (Inovise Medical) remote patient monitoring is a noninvasive recording device that measures cardiac acoustic biomarkers (CABs) such as electromechanical activation time (also known as EMAT, or the time from QRS onset to the first heart sound interval) and the third heart sound strength using automated acoustic cardiography [38]. CABs are analyzed using a machine learning algorithm to ultimately predict progression of HF. A randomized control trial recently tested Audicor CAB-guided HF management versus symptom-guided management and demonstrated a significant reduction in rehospitalization for HF and total mortality during 12-month follow-up (*p* = 0.0095) [39].

BodiGuide Edema Monitor (BodiGuide Inc.) is another noninvasive, remote monitoring device worn on the ankle that uses position, orientation, and circumference sensors to measure interstitial fluid retention and predict impending HF decompensation. A recent open pilot study suggested feasibility in recording and identifying increasing trends in ankle circumference using the BodiGuide system [40].

CardioTag (Cardiosense Inc.) is a noninvasive RM wearable sensor that utilizes seismocardiogram (measuring of body vibrations induced by the heart), electrocardiogram, and photoplethysmogram (measuring of volumetric variations of blood circulation using infrared light) signals combined with machine learning algorithms to estimate pulmonary capillary wedge pressure [41]. A proof-of-concept study analyzed 20 patients with HF undergoing a right heart catheterization and vasodilator challenge and developed a population regression model to estimate changes in pulmonary capillary wedge pressure with changes in the CardioTag’s signals [42]. The study demonstrated reasonable accuracy for the validation set (root-mean-square error = 2.9 mm Hg; R2 = 0.95). Similar to CardioTag, the Acorai Heart Monitor and SAVE Sensor System is a multisensor system that combines seismocardiography, photoplethysmography, phonocardiography, and electrocardiography sensors into a handheld device. The device is placed on the upper chest for 2 min while lying flat and uses machine learning techniques to estimate intracardiac measurements, including pulmonary capillary wedge pressure. An observational study compared the correlation between estimated mean pulmonary artery pressure (mPAP) and right heart catheterization mPAP among 281 patients, demonstrating a correlation of 0.75 (r2 = 0.55) [43].

VitalPatch (Vital Connect) is a wearable sensor placed on the chest using an adhesive that collects continuous ECG waveform, 3-axis accelerometry, skin impedance, skin temperature, and information on activity and posture and pairs via Bluetooth to a smartphone which ultimately transmits to a cloud analytics platform (PhysIQ) [44]. The platform then uses a daily average of the data and machine-based learning to develop a personalized algorithm to predict HF rehospitalization. LINK-HF examined the performance of this machine learning system among 100 subjects with class II–IV HF and recent HF admission [44]. The system predicted HF hospitalization with 76 to 88% sensitivity and 85% specificity, with the median time between initial alert and readmission of 6.5 (4.2–13.7) days.

The Wearable Congestive HF Management System (WCHFS, also known as SimpleSENSE (Nanowear)) is a wearable undergarment that utilizes multiple sensors to develop an algorithm capable of predicting worsening heart failure. NanoSENSE is an ongoing multicenter, prospective, observational study which aims to enroll up to 500 subjects with NYHA class II–IV HF and recent HFH and collect data on at least 150 heart failure hospitalizations (NCT03719079). NanoSENSE is purely observational and will use these data to develop and validate a multiparameter algorithm for the detection of an impending HF event.

The Sensinel System (Analog Devices) is a wearable, HF RM device that measures multiple clinical parameters, including thoracic impedance, single-lead ECG, heart sounds, respiratory rate, skin temperature, and posture [45]. The Sensinel System has predefined thresholds that, when met, trigger alerts to the clinical care team. Sensinel is not currently FDA approved, and a clinical trial is currently being designed to assess its efficacy in reducing HFH.

The SCALE-HF 1 study (Surveillance and Alert-Based Multiparameter Monitoring to Reduce Worsening Heart Failure Events) is an ongoing, observational study designed to evaluate the sensitivity and unexplained alert rate of the Bodyport cardiac scale-derived composite index (Bodyport) in predicting HF events, defined as urgent, unscheduled clinic, emergency department, or hospitalization for worsening HF among 300 patients with chronic HF and recent decompensation [46]. The composite index is developed solely from noninvasive hemodynamic biomarkers measured by the cardiac scale, using electrocardiography, ballistocardiography, and impedance plethysmography. The cardiac scale is a physical platform that patients stand on for 20 to 30 s while measurements are obtained. It then compares the measurements to known measurements and trends for a patient and uses cellular network connectivity for the transmission of data and, ultimately, integration of data into the electronic health record for clinical review.

### 2.7. Real-World Implementation of an Effective RM Algorithm: A Clinical How-To

Real-world clinical experience with RM devices is rather limited. Cited barriers to implementation include determining which parameters to monitor, determining how to integrate and manage exponential numbers of data within the realms of the existing clinical structure, and determining how to respond to data trends and alerts. Equally critical to widespread uptake is identifying patients who would respond to RM devices and implementing RM devices within the confines of the existing clinical infrastructure and reimbursement models. We provide a real-world example of implementing RM and use it to discuss these relevant considerations.

**Figure 3:**
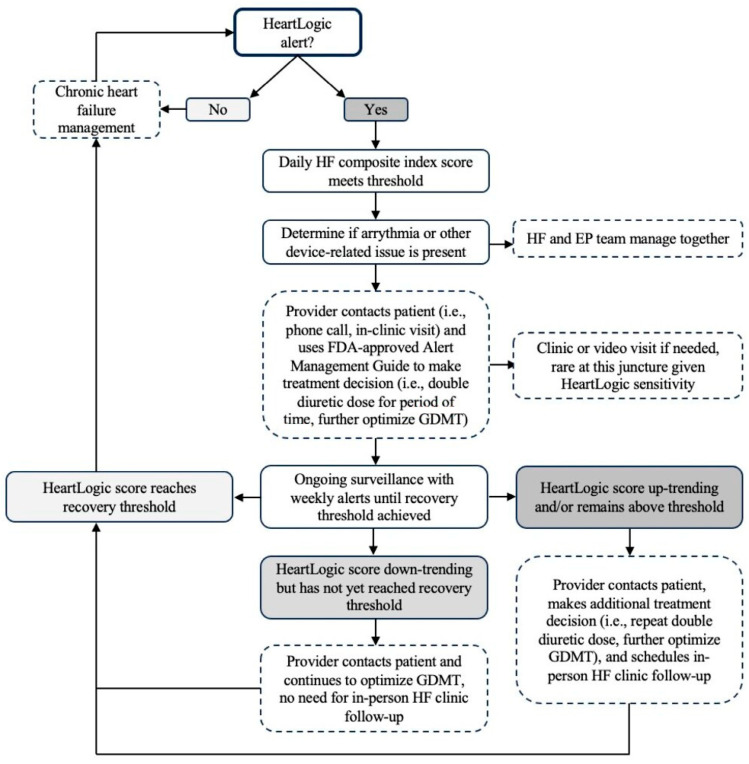
Flow Chart of HeartLogic Alert Management. HF, heart failure; FDA, Food and Drug Administration; GDMT, guideline-directed medical therapy.

Artificial intelligence, specifically using machine learning methods to identify HF predictive parameters using continuous data trends, is a promising method to incorporate and handle growing numbers of RM data. The HeartLogic algorithm, as previously discussed within the context of the MULTISENSE study, is one example of machine learning with demonstrated effectiveness [32]. The algorithm uses Boston Scientific ICD- and CRT-D-measured parameters to generate a daily HF composite index score. Alerts are triggered when the index score exceeds a specific threshold, signifying a patient is high risk for an HF event. Figure 3 demonstrates a flow chart of how alerts are clinically managed. Providers automatically receive this initial alert and receive weekly alerts thereafter until a recovery threshold is reached. In response to alerts, providers are required to make a response (guided by an FDA-approved Alert Management Guide), such as a patient phone call or in-clinic visit, adherence reinforcement, medication change, or documentation justifying why no change was made. This ensures provider liability for abnormal patient data. Once data normalize, RM returns to routine remote interrogations or in-clinic interrogations.

Like the HeartLogic algorithm, the TriageHF algorithm is a validated, FDA-approved HF-risk-prediction tool [47]. The algorithm uses Medtronic ICD- and CRT-D-measured parameters to generate a patient-specific assessment of risk level (high, medium, or low risk) for HFH in the next 30 days. A validation study of 921 patients demonstrated that compared to individuals within the low-risk group, individuals within the high-risk group were 10 times more likely to have an HFH in the next 30 days (adjusted HR 10.0; 95% CI 6.4–15.7; *p* < 0.001), while those in the medium-risk group were 2.1 times more likely to have an HFH in the next 30 days (adjusted HR 2.1; 95% CI 1.3–3.4; *p* = 0.001) [47]. The TRIAGE-HF trial demonstrated that high-risk heart-failure-risk status had good predictive accuracy of worsening HF symptoms [48]. Further, the TriageHF risk metric was predictive on all 60 days prior to an HF readmission (*p* < 0.001), while the risk metric declined significantly one week postdischarge in patients without HF readmission (*p* < 0.001) [49]. For the purpose of this implementation how-to, we focus on the HeartLogic algorithm; however, we have also adapted the TriageHF algorithm for use in our RM clinic using the same processes detailed below.

**Figure 4:**
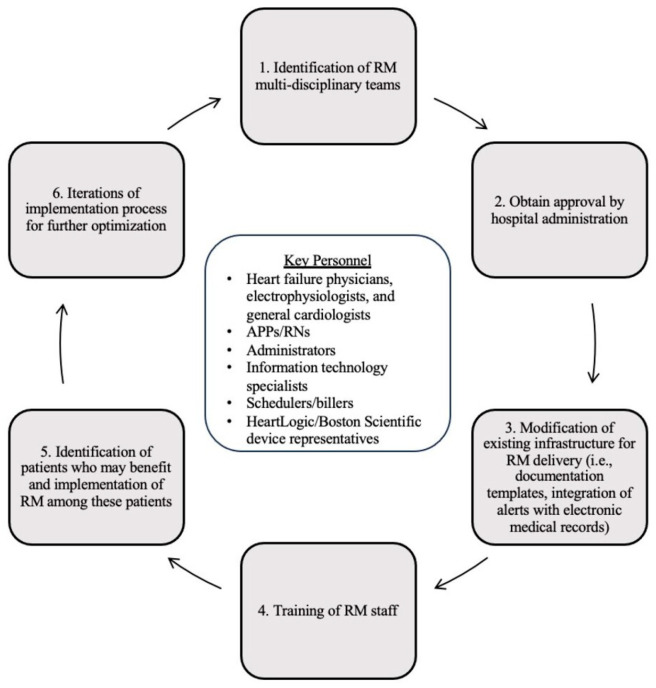
Remote Monitoring Implementation Process. APPs, advanced practice providers; RNs, registered nurses; RM, remote monitoring.

To implement the FDA-approved HeartLogic technology into clinical practice, we first established a remote monitoring clinic at our institution. Figure 4 depicts our remote monitoring implementation process. To do so, multidisciplinary teams were identified that included electrophysiologists, heart failure physicians, general cardiologists, advanced practice providers (APPs), registered nurses (RNs), device representatives, administrators, information technology (IT) specialists, and billers, each with an important role in the implementation process. Physicians collaborate in device selection, identify patients who may benefit from remote monitoring, and establish alert protocols and documentation templates. Heart failure cardiologists provide RM clinic oversight and perform the initial training of the RM team. Electrophysiologists perform device implantation and help manage EP-related alerts. APPs or similar complete weekly Merlin.net site logins, review all patients in alert status and their information in the EMR, contact patients, document the encounters, and relay the information to the cardiologist. The RM team adjusts diuretics and goal-directed medical therapy for those patients in alert status, as previously discussed and depicted in Figure 3. RNs and administrators assist with monthly billing and documentation. Device representatives enroll new ICD implants into the Latitude NXT Patient Management System, obtain login access for the RM team for patient management, and train the RM on how to navigate the Latitude patient site. Finally, our multidisciplinary team has a dedicated HF clinic that allows for urgent access if needed for patients who are symptomatic upon provider contact with a patient. In addition to these personnel needs, basic infrastructure is needed for RM. On the patient side, patients need access to WiFi for the uploading of data to the Merlin.net Patient Care Network, and the RM team needs access to the Latitude NXT Patient Management System. Our RM team works with third party vendors and our hospital’s IT specialists to integrate the Merlin patient data and the hospital EMR, allowing data, documentation, and billing to all exist in one area. While these personnel and infrastructure needs may seem resource-intensive, this process made use of mostly pre-existing clinical staffing and infrastructure outside of the HeartLogic technology and associated patient platforms. Further, based on previously presented data (Table 1), RM possesses the capability to significantly offload the clinical burden of HFH. Our estimates also suggest that RM can generate significant revenue based on a well-defined reimbursement model, per below.

Part of the implementation process included developing a reimbursement model for device implantation, interrogations, alerts, and associated telephone and in-person clinic visits. For implantation, the initial EP reimbursement remains unchanged as the HeartLogic algorithm is embedded in standard Boston Scientific CIED devices (same applies to the TRIAGE-HF algorithm for the Medtronic devices). For patients with a pre-existing Boston Scientific/Medtronic CIED, we process RM enrollment and billing in two separate ways depending on alert status frequency. Patients frequently in alert status are asked to enroll in monthly billing, while those not frequently in alert status are contacted through mail and asked to enroll in monthly billing through an opt-in approach. For patients without a pre-existing CIED, we discuss the HeartLogic/TriageHF technology and RM clinic with them at the time of consideration of new ICD implantation and have them enrolled in monthly billing through an opt-out approach. Monthly diagnostic device evaluation consists of two components: (1) a CPT code for the procession portion (CPT 93297), and (2) a CPT code for the technical portion (CPT G2066). Physician EMR documentation and attestation is required for both. Of note, RM billing can occur as alert-based billing (for patients not enrolled in monthly billing); however, our RM team has not yet undertaken this. Also, it is important to note that billing for HF management (monthly) and billing for EP device remote interrogation (every 91 days) cannot be reported in the same 30-day monitoring period. Finally, if a patient needs to be seen in an HF clinic due to an alert, this is a separately billed event. These revenue opportunities were used to leverage the clinical need for establishing an RM clinic at the hospital administration level, allowing for dedicated personnel and resources.

## 3. Discussion

RM represents a promising approach to monitoring and caring for patients with HF. The scope of RM devices is broad, including implantable monitors, wearable devices, and advanced technological capabilities of already existing CIEDs. This review of the current RM literature suggests that RM for patients with class II–IV HF is overall safe, accurate, and has potential for reducing HFH and all-cause mortality. However, the RM clinical trials examined varied greatly in the types of information monitored—from single parameters such as pulmonary artery pressure or inferior vena cava dimensions to multiparameter, CIED-based algorithms. Trials also varied in prerequisites for the selected patient population (i.e., prior HFH or not, severity of HF), the frequency of data transmissions and clinician review, and perhaps most importantly, how exponential numbers of data were managed and acted upon. These differences likely account for a few trials finding null treatment outcomes. As such, the data regarding the ideal RM approach are likely yet to be established, underscoring a key area of ongoing research.

Perhaps equally important to establishing the ideal RM approach is establishing a real-world implementation model. Here, we discussed the successful and pragmatic implementation of an RM program at a large academic center. Our approach highlights the capabilities of evidence-based machine learning to manage exponential numbers of patient data; the importance of utilizing existing clinical personnel, infrastructure, and reimbursement models; and the necessity of multidisciplinary teams. This practical approach emphasizes the highly adaptable nature of RM for HF to fit a multitude of healthcare structures and settings. However, as uptake of RM continues to increase, the number of patient data will grow exponentially, making the automation of data collection, processing, documentation, and billing critically important.

For data collection and processing, most RM devices highlighted in this review collect patient data in real-time, automatically triggering alerts to the provider once prespecified patient thresholds were reached. This alert triggering is entirely dependent upon accurate recognition of abnormal physiological trends. As our experience with the HeartLogic algorithm demonstrated, artificial intelligence and machine learning methods are particularly well equipped to analyze continuous, multimodal data; recognize deviant values over time; and develop accurate prediction models for HF decompensation.

Equally imperative is the ability to document data and alerts in a quickly viewable and secure manner. While data documentation and viewing by providers in the examined studies varied depending on the RM device and its associated platform, software to integrate all device data and interrogations into one place already exists and is rapidly evolving. For example, software platforms such as PaceMate or Murj allow for the automatic incorporation and billing of RM data with major electronic health record (EHR) systems. This integration process helps to eliminate the manual entry and uploading of patient data to the EHR, give access to patient data in real-time, automate billing with detailed financial transaction configurations, and reduce administrative costs and provider time burden, all of which increase the efficiency of patient care and reduce downstream healthcare costs.

There are a few limitations to this review. For one, this is not a systemic review as defined by the PRISMA checklist. As such, this review did not have set inclusion criteria; did not explore possible causes of heterogeneity among study results; and did not provide measures of cumulative effectiveness, bias, or certainty. Similarly, we recognize this review likely does not include all possible RM devices and technology currently in existence. Rather, the purpose of this review is primarily to highlight the scope of RM in clinical practice through a sampling of key devices and techniques and to provide a framework for RM implementation based on first-hand experiences.

Ultimately, the ongoing automation of RM medical care is proving indispensable, with further development and refinement needed to maximize RM capabilities. At this time, we recognize that the automation capabilities and necessary RM infrastructure required to start an RM clinic—two key pillars to RM implementation—may not be ubiquitous at smaller or nonacademic medical centers, representing a notable barrier for widespread uptake and equitable delivery of RM. However, in many ways, RM can expand the catchment areas of resource-rich medical systems, reaching patients that otherwise would not have such specialized HF care or monitoring.

Overall, this review highlights the potential of RM devices to improve HF outcomes, the feasibility of implementing an RM clinic in real-world clinical practice, and the needed areas of ongoing research to fully leverage RM capabilities for HF management.

## Figures and Tables

**Table 1:** Overview of key clinical trials in implantable device-based RM.

Device	Study	Patient Population	Data Collection and Storage	Frequency of Data Monitoring and Data Management	Outcome
Implantable pulmonary artery pressure monitoring devices					
CardioMEMS	CHAMPION (2007–2010)	550 patients with NYHA class III HF across 64 sites in the U.S.	- CardioMEMS pillow collected data/portable electronic unit - Data transmitted to secure Internet-based database for physician review	- Recorded PAP daily - Prespecified guidelines based on PAP measurements	- Reduction in HFH but not all-cause mortality at 6 months
	Postapproval study (2014–2017)	1200 patients with NYHA class III HF and prior HFH within 12 months across 104 sites in the U.S.		- Recorded PAP daily - Prespecified guidelines based on PAP measurements	- Reduction in HFH at 12 months
	GUIDE-HF (2018–2022)	1000 patients with NYHA class II–IV HF and prior HFH within 12 months or elevated BNP across 118 sites in the U.S. and Canada		- Recorded PAP daily - Specific interventions at discretion of treating provider but aimed to achieve PAP goal ranges as in CHAMPION trial	- Reduction in primary endpoint of HFH/urgent visits and all-cause mortality at 12 months
	MEMS-HF (2016–2020)	234 patients with NYHA class III HF across 31 sites in Europe		- Recorded PAP daily - Reviewed at least weekly or sooner if automatic alert triggered	- Freedom from device- or system-related complications and from sensor failure at 12 months - Reduction in HFH at 12 months
	MONITOR-HF (2019–2022)	348 patients with NYHA III and prior HFH across 25 sites in the Netherlands		- Record PAP daily - Prespecified guidelines based on evidence of excess intravascular volume or resistance	- Freedom from device- or system-related complications and from sensor failure at 12 months - Improvement in quality of life (by Kansas City Cardiomyopathy Questionnaire) and reduction in HFH at 12 months
Cordella endotronix	SIRONA (2017–2019)	15 patients with NYHA class III HF in Europe	- Combined with a Wireless handheld reader (myCordella Patient Reader) and tablet, which collect and transmit data to a web-based Patient Management Portal (secure cloud-based platform) for review by clinical team	- Recorded PAP daily in addition to vital signs	- No device- or system-related complications or sensor failure - Met primary efficacy endpoint of mean PAP at 90 days
	SIRONA II (2019–2021)	81 patients with NYHA class III HF in Europe			- Met primary efficacy endpoint of accuracy, comparing the PA sensor mean PAP measurements with RHC PAP measurements - Minimal device- or system-related complications - No sensor failure
	PROACTIVE-HF (2020-estimated 9/2023)	456 patients with NYHA class III HF currently enrolled across 79 sites in the U.S. and Europe		- Recorded PAP daily in addition to vital signs and symptoms - Reviewed at least once every 4 days - The 7-day mean PAP along with other data used to make guideline-based decisions	- Ongoing, estimated study completion March 2026 - Freedom from device-/system-related complication and from pressure sensor failure - HFH or all-cause mortality at 6 months
Implantable left atrial pressure monitoring devices					
Vectorius V-LAP	VECTOR-HF (2019-estimated 12/2023)	24 patients with NYHA class III HF across two sites in Europe	- Combined with an external reader that transmits data to a secured Cloud Storage database	- Recorded LAP daily - Alerts triggered to patient and provider when LAP is out of optimal range	- Ongoing trial - Data to date suggest agreeability with wedge-pressure measurements and effectiveness in improving NYHA functional class status
Implantable inferior vena cava monitoring devices					
FIRE1	FUTURE-HF (NCT04203576) (2019-estimated 8/2023)	Goal enrollment of 50 patients with HF and prior HFH within 6 months across 10 sites in Europe	- Combined with an external detection belt that transmits data for review by clinical team	- Recorded IVC dimensions daily	- Ongoing trial - Primary safety endpoint is procedural success and freedom from FIRE1 sensor complications at 3 months - Primary technical endpoint is signal acquisition at 3 months
Implantable cardiac monitors					
LUX-Dx ICM	TRENDS (NCT04790344) (2021-estimated 5/2026)	Goal enrollment of 525 patients with NYHA class II–III HF across 74 sites in the U.S.	- Combined with a patient app that transmits data to the Latitude Clarity Data Management System for review by clinical team	- Loop recorder that records heart rhythms 24/7	- Ongoing trial - Primary outcome of comparing diagnostic sensor data with reference clinical testing data and heart failure decompensation events
Reveal LINQ ICM	ALLEVIATE-HF (NCT04452149) (2020-estimated 11/2024)	Goal enrollment of 700 patients with NYHA class II–III across 59 sites in the U.S.	- Combined with a transmitter or smartphone app that downloads and transmits data securely for review by clinical team	- Loop recorder that records heart rhythms 24/7 - Diagnostic-based risk stratification algorithm	- Ongoing trial - Primary outcomes of safety and efficacy of a patient management pathway

BNP, brain natriuretic peptide; HF, heart failure; HFH, heart failure hospitalization; ICM, implantable cardiac monitors; IVC, inferior vena cava. LAP, left atrial pressure; NYHA, New York Heart Association; PAP, pulmonary artery pressure; RM, remote monitoring; U.S., United States.

**Table 2:** Overview of key clinical trials in CIED-based RM.

Device	Study	Patient Population	Data Collection and Storage	Frequency of Data Monitoring and Data Management	Outcome
Cardiovascular Implantable Electronic Device (CIED) Monitoring					
CIEDs	FAST (2003–2008)	156 patients with NYHA class III–IV HF and a Medtronic CIED	- Software was downloaded onto the device to measure and store impedance measurements	- Recorded daily changes in intrathoracic impedance - Both clinicians and patients did not have access to data during the trial	- Increased sensitivity and lower unexplained detection rate in detecting HF events compared to weight monitoring
	OptiLINK HF (2008–2014)	1002 patients with NYHA class II–III HF, a Medtronic CIED, prior HFH within 12 months, recent diuretic treatment, or elevated BNP across 1 site in Europe	- Devices set to automatically transmit fluid index alerts in treatment arm	- Continuous data monitoring - A protocol-specified intervention algorithm was followed if alert triggered	- No difference in primary composite endpoint of all-cause death and cardiovascular hospitalization
	PARTNERS-HF (2004–2008)	694 patients with NYHA class III–IV HF and Medtronic CIED across 93 sites in the U.S.	- Collection of standard CIED-measured parameters	- Continuous data monitoring, but device interrogations occurred monthly	- Diagnostic algorithm successfully determined predictors of HFH
	REM-HF (2011–2014)	1650 patients with NYHA class II–IV HF with a CIED	- Collection of standard CIED-measured parameters - Data stored on electronic record	- Continuous data monitoring, but device interrogations occurred weekly - Formalized procedural handbook guided clinical management	- No difference in primary composite outcome of all-cause death or unplanned cardiovascular hospitalization
	MULTISENSE (2010–2014)	900 patients with NYHA class II–III HF with a CIED across 99 sites in Europe, Asia, and the U.S.	- Software was downloaded onto device that allowed data collection of HeartLogic algorithm parameters - Data downloaded at in-person visits or remotely using LATITUDE (computer platform) transmissions	- Continuous data monitoring used to give a daily HeartLogic index value	- Met prespecified, coprimary endpoint thresholds for sensitivity of detecting HF exacerbations and unexplained alert rate
	SELENE HF (2012–2017)	918 patients with NYHA class II–III HF across 34 sites in Europe	- Collection of standard CIED measured parameters	- Continuous data monitoring used to give a Seattle Heart Failure Model score	- Predictive algorithm successfully determined predictors of HFH
	TRUST (2005–2009), ECOST (2007–2010), IN-TIME (2007–2012)	2405 patients with HF and varying inclusion criteria across 171 sites in Europe and the U.S.	- Small, portable patient device receives data and transmits them automatically over a mobile phone, which links to the Home Monitoring Service Center on a secure site for review by clinical teams	- Daily transmissions of cumulative and last-saved diagnostic data	- Meta-analysis of three trials - Pooled effect demonstrated reduction in all-cause mortality and composite outcome of all-cause mortality or HFH

CIED, cardiovascular implantable electronic device; HF, heart failure; HFH, heart failure hospitalization; NYHA, New York Heart Association; U.S., United States.

## Data Availability

Data sharing is not applicable to this article as no new data were created or analyzed in this study.
